# Short- and Long-Term Outcomes in Elderly Patients Following Hand-Assisted Laparoscopic Surgery for Colorectal Liver Metastasis

**DOI:** 10.3390/jcm12144785

**Published:** 2023-07-20

**Authors:** Ahmad Mahamid, Omar Abu-Zaydeh, Samar Mattar, Esther Kazlow, Dvir Froylich, Muneer Sawaied, Natalia Goldberg, Yael Berger, Eran Sadot, Riad Haddad

**Affiliations:** 1 Department of Surgery, Carmel Medical Center, Haifa 3436212, Israel; mahamidam@yahoo.com (A.M.); oabuzaydeh@gmail.com (O.A.-Z.); samar.mattar11@gmail.com (S.M.); ekazlow@gmail.com (E.K.); dvirfr7@gmail.com (D.F.); moneerswaed@gmail.com (M.S.); 2 The Ruth and Bruce Rappaport Faculty of Medicine, Technion-Israel Institute of Technology, Haifa 3200003, Israel; natalia.goldberg@gmail.com; 3 Department of Radiology, Carmel Medical Center, Haifa 3436212, Israel; 4 Department of Surgery, Rabin Medical Center, Petch Tikvah 4941492, Israel; yaelberger1@gmail.com (Y.B.); eransadot@gmail.com (E.S.); 5 Sackler School of Medicine, Tel Aviv University, Tel Aviv 6997801, Israel

**Keywords:** hand-assisted laparoscopic surgery, colorectal liver metastasis, outcomes, elderly patients

## Abstract

(1) Background: Hand-assisted laparoscopic surgery (HALS) has engendered growing attention as a safe procedure for the resection of metastatic liver disease. However, there is little data available regarding the outcomes of HALS for colorectal liver metastasis (CRLM) in patients over the age of 75. (2) Methods: We compare the short- and long-term outcomes of patients >75-years-old (defined in our study as “elderly patients” and referred to as group 1, G1), with patients <75-years-old (defined in our study as “younger patients” and referred to as group 2, G2). (3) Results: Of 145 patients, 28 were in G1 and 117 were in G2. The most common site of the primary tumor was the right colon in G1, and the left colon in G2 (*p* = 0.05). More patients in G1 underwent laparoscopic anterior segment resection compared with G2 (43% vs. 39% respectively) (*p* = 0.003). 53% of patients in G1 and 74% of patients in G2 completed neoadjuvant therapy (*p* = 0.04). The median size of the largest metastasis was 32 (IQR 19–52) mm in G1 and 20 (IQR 13–35) mm in G2 (*p* = 0.001). The rate of complications (Dindo-Clavien grade ≥ III) was slightly higher in G1 (*p* = 0.06). The overall 5-year survival was 30% in G1 and 52% in G2 (*p* = 0.12). (4) Conclusions: Hand-assisted laparoscopic surgery for colorectal liver metastasis is safe and effective in an elderly patient population.

## 1. Introduction

Colorectal cancer (CRC) is the third most common malignancy worldwide. It accounts for 1.8 million newly diagnosed cases and 900,000 deaths annually, with metastatic tumors being the most common cause of death. The majority of CRC cases are observed in patients over the age of 65 [1].

Colorectal liver metastases (CRLM) occur in almost half of all patients with colorectal cancer and surgical resection for colorectal liver metastases has a 60% long-term survival rate at 5 years [2,3].

Over the past 3 decades, the development of minimally invasive surgery revolutionized the landscape of abdominal surgery, especially surgery of the hepatobiliary tract. Minimally invasive liver resection has become a standard practice and a good alternative to open liver resection in many instances. In 2008, The Louisville Consensus Conference divided hepatic laparoscopic procedures into three main categories: pure laparoscopy, hand-assisted laparoscopic surgery (HALS), and a hybrid technique [4]. The advantages of HALS for the surgeon are improved intraoperative bleeding control, detection of deeper intraparenchymal lesions, and better exposure of difficult tumor locations. Studies have shown HALS for CRLM to have similar outcomes to pure LLR and open liver surgery, including the early and late oncological outcomes, blood loss, conversion to open rate, operative time, overall morbidity and mortality, and length of hospital stay [5]. However, there is very little data regarding the safety and feasibility of HALS for CRLM in patients over the age of 70-years [6]. With an increased patient load in the geriatric age group, it has become increasingly relevant to assess whether elderly patients might benefit from a minimally invasive surgical approach. there is no constant definition for elderly age in the literature in the context of liver resection. few studies used 70 years as the cutoff [6], and others divided their cohort into 3 subgroups based on age (70–74, 75–79, and >80-years old) [2]. We decided to use age 75 as the cutoff to define elderly in our cohort. The aim of this study was to examine the perioperative and long-term outcomes of HALS for CRLM in patient over the age of 75.

## 2. Materials and Methods

We selected patients for our study from the surgical databases of the Rabin Medical Center (Petah Tikva, Israel) & the Carmel Medical Center (Haifa, Israel). These were patients who underwent HALS for CRLM between December 2004 and January 2019. The patients’ records were examined retrospectively. Criteria for inclusion to our study included pathology results, demographics, surgical history, and oncologic follow-up records. Patients ≥75 years old were assigned to group 1 (G1) and patients <75 years old to group 2 (G2). Primary outcomes were defined as the perioperative and histological results, and the secondary outcomes were defined as the overall survival over the follow up period.

The study was approved by the institutional review board (IRB) of both institutions. Surgical indications were determined during a weekly multidisciplinary conference. Pre-operative workup included biochemical analysis for blood count, chemistry, and tumor markers. The patients also underwent imaging which included MRI, CT, & PET-CT. This facilitated the indentification of tumors, their size, number, location, and interrelation with the vascular and biliary anatomy.

Metastasis with vascular contact were considered high risk for HALS approach and those patients underwent conventional laparotomy for liver resection. Patients with synchronous metastasis treated with liver first approach, colon first or combined approaches were done in case of complication of the primary tumor (bowel obstruction, perforation, bleeding). All patients underwent standard evaluation for major surgery by an attending anesthesiologist. They were informed about the procedure by the attending surgeon, including the risks and benefits, and written consent was obtained before surgery.

### 2.1. Surgical Technique

The approach for HALS for CRLM was performed as described by Sadot et al. [7]. In summary, patients were placed in a supine position. The surgeons inserted two 12 mm and one 5 mm trocar in the upper abdomen at the midline. A hand-assisted device was placed in the right abdomen. We used a supraumbilical cut to establish pneumoperitoneum with a 12-mm port in the majority of patients. However, taking into consideration the possibility of peritoneal adhesions, the surgeons performed a right abdominal horizontal incision in any patient with a history of abdominal surgery. Any adhesions were lysed. Following that, the hand port and a 12 mm trocar were inserted. CO_2_ gas was used to generate a pneumoperitoneum with a pressure of 12–15 mmHg. The abdomen was then explored visually with a 30° laparoscope. Meticulous laparoscopic intraoperative ultrasonography of the liver was routinely performed. Using the LigaSure™ device, liver mobilization and lysis of adhesions was performed. Biopsies and resections of the liver were performed using LigaSure™, Endo GIA™ staplers, and Cavitron Ultrasonic Surgical Aspirator. Following the resection, careful examination was performed to check for bile leakage and/or bleeding. An abdominal drain was placed through one of the port sites. Following the deflation of the pneumoperitoneum, the abdomen was closed. The specimens were sent to the pathology department for inspection of the surgical margins.

We defined metastasis by the presence of tumor cells at the time of diagnosis or during post-surgical follow up. Blood loss was estimated using the volume of blood aspirated from the abdominal cavity during the procedure. Operative time was defined as the time elapsed from the skin incision until closure. Postoperative hospital stay was defined as the number of hospitalized days from the day of operation until the day of discharge, inclusive. We used the Clavien-Dindo grading system to characterize any post-operative complications occurring within 30 days of surgery [8]. Tumor size and resection margins were determined according to the pathological reports from the permanent sections of tissue samples. Any specimen with no tumor cells seen on a microscopic level were defined as R0.

After discharge, the patients were followed by our multidisciplinary team during the first month, every 4 months for the first 2 years, and twice a year thereafter. Follow-up included clinical examinations, blood work-up including carcinoembryonic antigen (CEA), and spiral CT of the chest-abdomen or PET-CT as indicated.

### 2.2. Statistical Analysis

All statistical analyses were performed using IBM statistics (SPSS) vs. 24. Continuous variables were summarized with mean ± SD or median & IQR, as appropriate. Categorical variables were presented as numbers and proportions. Disease free (DFS) and overall survival (OS) were estimated using Kaplan-Meier curves and compared between groups by the log-rank test. *p* < 0.05 was considered statistically significant.

## 3. Results

From December 2004 until January 2019, HALS was performed in one hundred and forty-five patients for CRLM. Twenty-eight patients (19%) were ≥75 years old, and were assigned to group 1. Patient demographics and tumor characteristics are summarized in Table 1. The median age of group 1 was 80 (IQR 77–83) years and 68% were males. In 47% of the patients, the right colon was the origin of the primary tumor and in 39% of patients, the left colon was the origin of the primary tumor. 75% of patients had single liver metastasis, with a median size of 32 (IQR 19–52) mm for the largest metastasis. 82% had low calculated clinical risk score, and 53% completed neoadjuvant therapy.

Perioperative characteristics and outcomes are described in Table 2. 21% underwent formal lobectomy. Anterior segmental resection was performed in 43%.

The median operative time was 168 (IQR 147–235) minutes. In three patients, the surgery was converted to open resection. 29% required intraoperative blood transfusion. There was no patient mortality within the first 30 days post-resection. Surgical complications occurred in 10 patients (36%) (Dindo-Clavien grade ≥ III). Median hospital stay was 7 (IQR 5–10) days. R0 margins were achieved in 93% of the specimens. Adjuvant chemotherapy was successfully completed in 82% of the patients.

**Figure 1:**
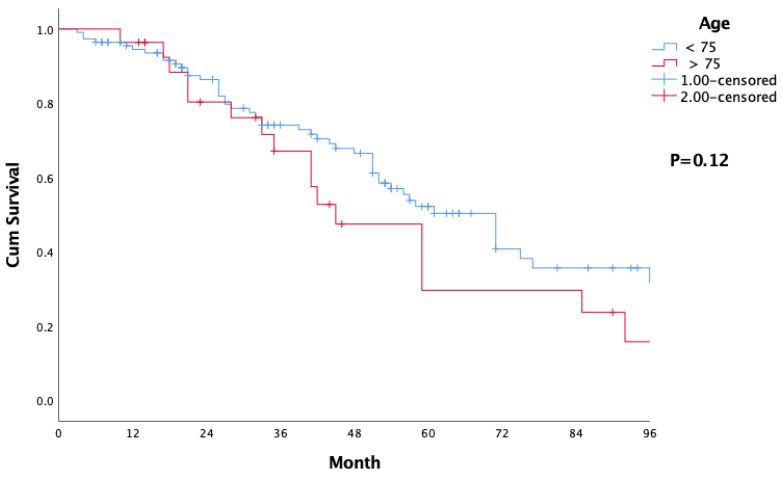
Comparison of overall survival between the elderly and the younger group.

The median follow-up period was 41 months (IQR 19–92), with median overall survival of 45 months (IQR 31–58). The overall survival at 1 year, 2 years, 3 years, and 5 years was 96%, 80%, 67%, and 30% respectively (Table 3, Figure 1).

### Comparison between Patients in G1 and G2

Group 1 consisted of 28 patients and was compared with a separate control group of 117 consecutive patients who underwent HALS for CRLM between December 2004 and January 2019, and were <75 years old (group 2, G2) (Table 1, Table 2 and Table 3).

There was no statistically significant difference between G1 and G2 in terms of gender, Fong clinical risk score, operative time, blood transfusion, hospital stay, adjuvant chemotherapy, 30-day mortality rate, and history of pervious abdominal surgery for the primary tumor (Table 1 and Table 2). In terms of conversion rates, there was no statistically significant difference between the groups. However, in 3 out of 28 patients (11%) in group 1, and 5 out of 117 (4%) in group 2 we had to convert due to technical difficulties and no progressing. No emergent conversion for bleeding and vital instability was needed. The most common site of the primary tumor was the right colon in G1, and the left colon in G2 (*p* = 0.05). 53% of patients in G1 and 74% of patients in G2 completed neoadjuvant therapy (*p* = 0.04). The median size of the largest metastasis was 32 (IQR 19–52) mm in G1 and 20 (IQR 13–35) mm in G2 (*p* = 0.001). The majority of G1 (75%) and a little less than half of G2 (47%) had one liver metastasis (*p* = 0.03) (Table 1). More patients in G1 underwent anterior segment resection compared with more posterior segmental resections in G2—43% vs. 39% and 18% vs. 49% respectively (*p* = 0.003). Patients in G1 tended to have more complications (Dindo-Clavien grade ≥ III) than patients in G2, without reaching statistical significance (36% vs. 19%) (*p* = 0.060). In G1 and G2, the R0 resection rate was 93% and 91%, respectively (*p* = 0.78) (Table 2). The overall 1-year and 5-years survival was 96% and 30%, respectively in G1, and 95% and 52%, respectively in G2 (*p* = 0.12) (Table 3, Figure 1).

## 4. Discussion

Our study suggests that HALS for CRLM in patients over 75 years old is safe and effective, with comparable outcomes to other surgical approaches.

Advances in medical care has led to an increasing number of patients who are now in their eighth and ninth decades. Many of these patients are having increasingly complex medical conditions [9,10].

Colorectal liver metastasis (CRLM) occurs in more than 50% of patients with colorectal cancer (CRC) [11]. Moreover, the majority of patients who have been diagnosed with CRC are older than 65 years and given the worldwide trend in population aging, more elderly patients will be presenting with potentially resectable CRLM [12,13].

Minimally invasive surgery (MIS) for liver neoplasms has become increasingly widespread. There are three approaches to minimally invasive hepatic surgery; standard laparoscopy, hand-assisted laparoscopy, and a combined approach. In the standard laparoscopic procedure, the entire operation is completed through laparoscopic ports. In hand-assisted laparoscopic surgery (HALS) a hand port is used to assist the procedure. Lastly, in the hybrid technique, the patient undergoes standard laparoscopy or HALS, but the liver resection is done through a mini-laparotomy incision [4].

Three consensus guidelines (Louisville [4], Morioka [14] and Southampton [15]) on laparoscopic liver resection (LLR) estimates that pure laparoscopic liver resection, HALS, and the hybrid technique appear to have equivalent outcomes and are simply a matter of surgeon preference and case selection.

In a previous study, we found that HALS is a safe and effective approach in a specific subset of patients with colon cancer and liver disease. Results are comparable to the pure laparoscopic and open techniques [5]. We believe this is the first study to evaluate treatment outcomes of patients ≥75-years old who have underwent HALS for CRLM. There have been a few reports about minimally invasive surgery in an elderly patient population without sub-analyses for the HALS group to date [6].

The results of this study suggest that HALS for CRLM in patients over the age of 75 is safe, effective, and does not increase the rates of morbidity or mortality. We had a lower mortality rate compared with a series of open hepatectomies [16,17], and the same rates compared with pure laparoscopic hepatectomies [18]. Our complication rate was slightly higher in the elderly patient group (*p* = 0.06). However, this still compares well with reports of both pure laparoscopic and traditional open hepatectomy [16,17,18]. The median operation time was approximately 2.8 h in both groups with no significant differences. There was no statistically meaningful difference between the groups in terms of the conversion rates, and the main reason to convert was due to technical difficulties and a lack of progression. No emergent conversion for bleeding and vital instability was needed.

The traditional open liver resection may increase the risk of cardiopulmonary complications through several mechanisms, such as painful limitation of the thoracic cage, resulting in a 50–60% reduction of the vital capacity and a 30% reduction in functional residual capacity [19]. HALS is less traumatic to the abdominal wall and typically results in decreased postoperative pain and early postoperative rehabilitation. It therefore may provide improved cardiopulmonary function recovery and shorten the hospital stay [20]. The hospital stay was comparable in the two groups, despite the fact that the conversion rate, blood transfusion rate, and major complications rate were relatively higher in group 1.

Radical (R0) liver resection is the gold standard for CRLM and offers longer survival [21,22,23]. In Martínez-Cecilia et al. study, which compared the perioperative and oncological outcomes of laparoscopic and open liver resection for colorectal liver metastases in the elderly, the R0 rate was 88% in both the laparoscopic and open approaches [2]. Nomi et al. provided an important bridge to this conclusion with results showing that laparoscopic surgery is indeed safe in elderly patients with an 84% R0 resection rate [18]. In this study, we found that using HALS combined with meticulous laparoscopic intraoperative ultrasonography, was safe and did not compromise the oncological outcomes in the elderly patients. This was evidenced by the 93% of R0 resections in group 1, compared with 91% in group 2.

Our results showed shorter median long-term overall survival in group 1 (45 months vs. 71 months, *p* = 0.12). We believe that shorter survival can be attributed to the difference between the median ages of the groups during liver resection (64 years vs. 80 years). This is likely due to more limited survival expectancy, and not to the surgical technique nor to the oncological causes. These results compare well with those reported by Martínez-Cecilia et al. Their 1-year, 3-year, and 5-year survival rates were 93%, 68%, and 43% respectively, vs. 96%, 67% and 30% respectively in our study [2].

The retrospective nature of our present study and the relatively small sample size confer some limitations. We believe that a multi-center prospective randomized control trial or using the propensity score matching method in a larger cohort will be the ideal study design to analyze the short- and long-term outcomes in elderly patients following hand-assisted laparoscopic surgery for colorectal liver metastasis.

## 5. Conclusions

This study demonstrates that HALS for CRLM in elderly patients is safe and effective with acceptable perioperative complications and long-term outcomes that are similar to those in younger patients. This suggests that advanced age itself should not be regarded as a contraindication for HALS for CRLM.

## Figures and Tables

**Table 1:** Baseline demographical, liver and colorectal characteristics.

	ALL (*n* = 145)	Young (<75) (*n* = 117)	Old (≥75) (*n* = 28)	*p* Value
Age (years) median	67 (59:73)	64 (57:70)	80 (77:83)	0.000
Gender				0.43
Male	89 (61%)	70 (60%)	19 (68%)	
Female	56 (39%)	47 (40%)	9 (32%)	
Primary tumor location				0.05
Right colon	40 (28%)	27 (23%)	13 (47%)	
Left colon	65 (45%)	54 (46%)	11 (39%)	
Rectum	35 (24%)	31 (27%)	4 (14%)	
Missing	5 (3%)	5(4%)		
Stage at colon diagnosis				0.47
II	39 (27%)	29 (25%)	10 (36%)	
III	31 (21%)	25 (21%)	6 (21%)	
IV	75 (52%)	63 (54%)	12 (43%)	
Liver tumor				
size of largest metastases (mm) median	20(13:35)	20(13:35)	32(19:52)	0.001
Number of Metastases				0.032
1	77 (53%)	56 (47%)	21 (75%)	
2	27(19%)	24 (21%)	3 (11%)	
>3	41(28%)	37 (32%)	4 (14%)	
Number, median	1(1:3)	2(1:3)	1(1:2)	0.019
Fong Clinical Risk Score				0.144
Low	100 (71%)	77 (68%)	23 (82%)	
High	41 (29%)	36 (32%)	5 (18%)	
Missing	4	4		
Neoadjuvant therapy	101 (70%)	86 (74%)	15 (53%)	0.039

**Table 2:** Perioperative and histological outcomes.

	ALL (*n* = 145)	Young (<75) (*n* = 117)	Old (≥75) (*n* = 28)	*p* Value
Type of liver resection				0.003
RT lobectomy	6 (4%)	4 (3%)	2 (7%)	
LT Lobectomy	5 (3%)	1 (1%)	4 (14%)	
LLT	22 (16%)	17 (15%)	5 (18%)	
Anterior segments (non-Anatomical)	58 (40%)	46 (39%)	12 (43%)	
Posterior Segments (non-Anatomical)	54 (37%)	49 (42%)	5 (18%)	
Conversion	8 (6%)	5 (4%)	3 (11%)	0.09
Operative Time (min) median	172 (125:233)	178 (125:240)	168 (147:235)	0.86
Blood Transfusion	34 (23%)	26 (22%)	8 (29%)	0.47
Complications	32(23%)	22 (19%)	10 (36%)	0.06
Hospital stay (days) median	6 (5:7)	5 (4:7)	7 (5:10)	0.22
surgical margins R0	132 (91%)	106 (91%)	26 (93%)	0.78
Adjuvant Chemotherapy	102 (70%)	79 (67%)	23 (82%)	0.14

RT, right; LT, left; LLT, Left lateral.

**Table 3:** Short- and long-term outcomes.

	ALL (*n* = 145)	Young (<75) (*n* = 117)	Old (≥75) (*n* = 28)	*p* Value
Median follow-up (months)		40 (23:56)	41 (19:92)	0.32
Overall survival				
Median (months)	59 (48:69)	71 (58:83)	45 (31:58)	0.12
12 (months)	94%	95%	96%	
24 (months)	85%	86%	80%	
36 (months)	73%	73%	67%	
60 (months)	47%	52%	30%	

## Data Availability

Data is unavailable.
